# Improved collateral flow and reduced damage after remote ischemic perconditioning during distal middle cerebral artery occlusion in aged rats

**DOI:** 10.1038/s41598-020-69122-8

**Published:** 2020-07-24

**Authors:** Junqiang Ma, Yonglie Ma, Ashfaq Shuaib, Ian R. Winship

**Affiliations:** 1 Neurochemical Research Unit, Department of Psychiatry, 12-127 Clinical Sciences Building, University of Alberta, Edmonton, AB T6G 2R3 Canada; 2 Neuroscience and Mental Health Institute, University of Alberta, Edmonton, AB Canada; 3 Division of Neurology, Department of Medicine, Faculty of Medicine and Dentistry, University of Alberta, Edmonton, AB Canada; 4 First Affiliated Hospital, Shantou University Medical College, Shantou, Guangdong China

**Keywords:** Diseases of the nervous system, Neuro-vascular interactions, Neurology, Preclinical research

## Abstract

Circulation through cerebral collaterals can maintain tissue viability until reperfusion is achieved. However, collateral circulation is time limited, and failure of collaterals is accelerated in the aged. Remote ischemic perconditioning (RIPerC), which involves inducing a series of repetitive, transient peripheral cycles of ischemia and reperfusion at a site remote to the brain during cerebral ischemia, may be neuroprotective and can prevent collateral failure in young adult rats. Here, we demonstrate the efficacy of RIPerC to improve blood flow through collaterals in aged (16–18 months of age) Sprague Dawley rats during a distal middle cerebral artery occlusion. Laser speckle contrast imaging and two-photon laser scanning microscopy were used to directly measure flow through collateral connections to ischemic tissue. Consistent with studies in young adult rats, RIPerC enhanced collateral flow by preventing the stroke-induced narrowing of pial arterioles during ischemia. This improved flow was associated with reduced early ischemic damage in RIPerC treated aged rats relative to controls. Thus, RIPerC is an easily administered, non-invasive neuroprotective strategy that can improve penumbral blood flow via collaterals. Enhanced collateral flow supports further investigation as an adjuvant therapy to recanalization therapy and a protective treatment to maintain tissue viability prior to reperfusion.

## Introduction

Ischemic stroke is a devastating cerebral disease that occurs when arteries supplying the brain are obstructed. Ischemia leads to insufficient nutrient and oxygen supply to meet metabolic demand of the brain, thus inducing the damage or death of brain cells. Cerebral collaterals are subsidiary vascular channels in the cerebral circulation which can sustain blood flow to ischemic tissue when principal vascular routes fail^1–6^. Blood flow through the collateral circulation is a primary determinant of the degree of ischemia in the penumbra, and thus a major predictor of infarct size and growth^8,3,7,9–12^. Cerebral collaterals can be classified as primary or secondary collaterals. The primary collaterals refer to the Circle of Willis, which allows blood flow exchange between anterior and posterior circulation and between hemispheres. Secondary collaterals include the pial or leptomeningeal collaterals^5^. Pial collaterals are anastomotic connections located on the cortical surface that connect distal branches of adjacent arterial networks (e.g. anterior cerebral artery (ACA)–middle cerebral artery (MCA); middle cerebral artery (MCA)–posterior cerebral artery (PCA) ACA–MCA; MCA–PCA^13,2,14,15^). MCA occlusion is the most common cause of ischemic stroke. Notably, when MCA occlusion occurs, pial collaterals become patent and can provide compensatory blood flow from the ACA and/or PCA to the ischemic penumbra via anastomotic connections with the MCA. However, collateral flow is time limited and can fail over time^16–21^. The failure of collaterals during stroke leads to the progression of the penumbra to irreversible ischemic infarct and reduces benefit from recanalization therapies^19,22–24^.

Intravenous recombinant tissue plasminogen activator (IV r-tPA) is the only FDA approved medical treatment of ischemic stroke, but requires administration within 4.5 h of symptom onset^25–28^. Although proven effective when administered within this therapeutic window, approximately half of patients treated with intravenous r-tPA do not benefit^29^. It is likely that insufficient collateral flow that leads to rapid tissue infarction accounts for the lack of benefit due to recanalization in many of these patients. Supporting this, multiphase CT angiography data from the ESCAPE (Endovascular treatment for Small Core and Anterior circulation Proximal occlusion with Emphasis on minimizing CT to recanalization times) trial identified a strong association between pre-treatment cerebral pial collaterals and favorable outcome after recanalization^30–32^. Similarly, patients with "slow-growing infarcts" due to good collateral circulation in the (DWI or CTP Assessment with Clinical Mismatch in the Triage of Wake-Up and Late Presenting Strokes Undergoing Neurointervention with Trevo) and DEFUSE3 (Endovascular Therapy Following Imaging Evaluation for Ischemic Stroke 3) trials benefitted from late thrombectomy (6 to 24 h after stroke onset), likely due to sufficient collateral circulation to maintain tissue viability prior to recanalization up to 24 h post stroke^33–42^. Rates of hemorrhagic transformation after recanalization are also reduced in patients with good collateral blood flow^4,43,44^. Strategies that can augment collateral blood flow to reduce expansion of the infarct core before recanalization treatment may therefore extend the time window for reperfusion interventions and allow more patients to benefit^3^.

Remote ischemic conditioning is a therapeutic process induced by series of repetitive, transient episodes of ischemia/reperfusion on a non-vital remote organ, such as limb, to provide endogenous protection to ischemia in vital organs such as the heart or brain^45,17,46,47^ (9,20). Remote ischemic perconditioning (RIPerC) involves starting treatment after the onset of vital organ ischemia but before reperfusion^17,48,49^. RIPerC induced by bilateral femoral occlusion (BFO) within 1 h post dMCAO is effective in preventing collateral collapse and reducing early ischemic damage in young adult rats^17^. However, aging is one of the primary risk factors for ischemic stroke and it is known that the brain of the elderly has reduced ischemic tolerance^50,9,51,52^. Preclinical studies have reported that increasing age is associated with rarefaction of cerebral collaterals, leading to insufficient ability to maintain blood flow during ischemia^9,50,3,16,53^. Notably, the hemodynamic evolution of the pial collateral circulation is also influenced by aging. Aged rats exhibit rapid and more severe failure of pial collaterals relative to young rats, and significantly greater volumes of early ischemic damage^16,53^. Whether RIPerC can prevent the collapse of collateral flow in aged rats is not known. Given that the cardioprotective effects of RIPerC are age dependent, it is important to confirm protective effects on collateral circulation and ischemic injury in aged animals that more closely model the clinical population^54^. To provide further evidence in support bench to bedside translation of RIPerC by directly demonstrating protection of collateral flow, we examined hemodynamic changes in pial collateral vessels after RIPerC treatment in aged rats. Our findings demonstrated that RIPerC induced by BFO 1 h after distal middle cerebral artery occlusion (dMCAO) is effective in reducing early ischemic damage and improves collateral blood flow by preventing collateral narrowing in aged animals.

## Results

**Figure 1:**
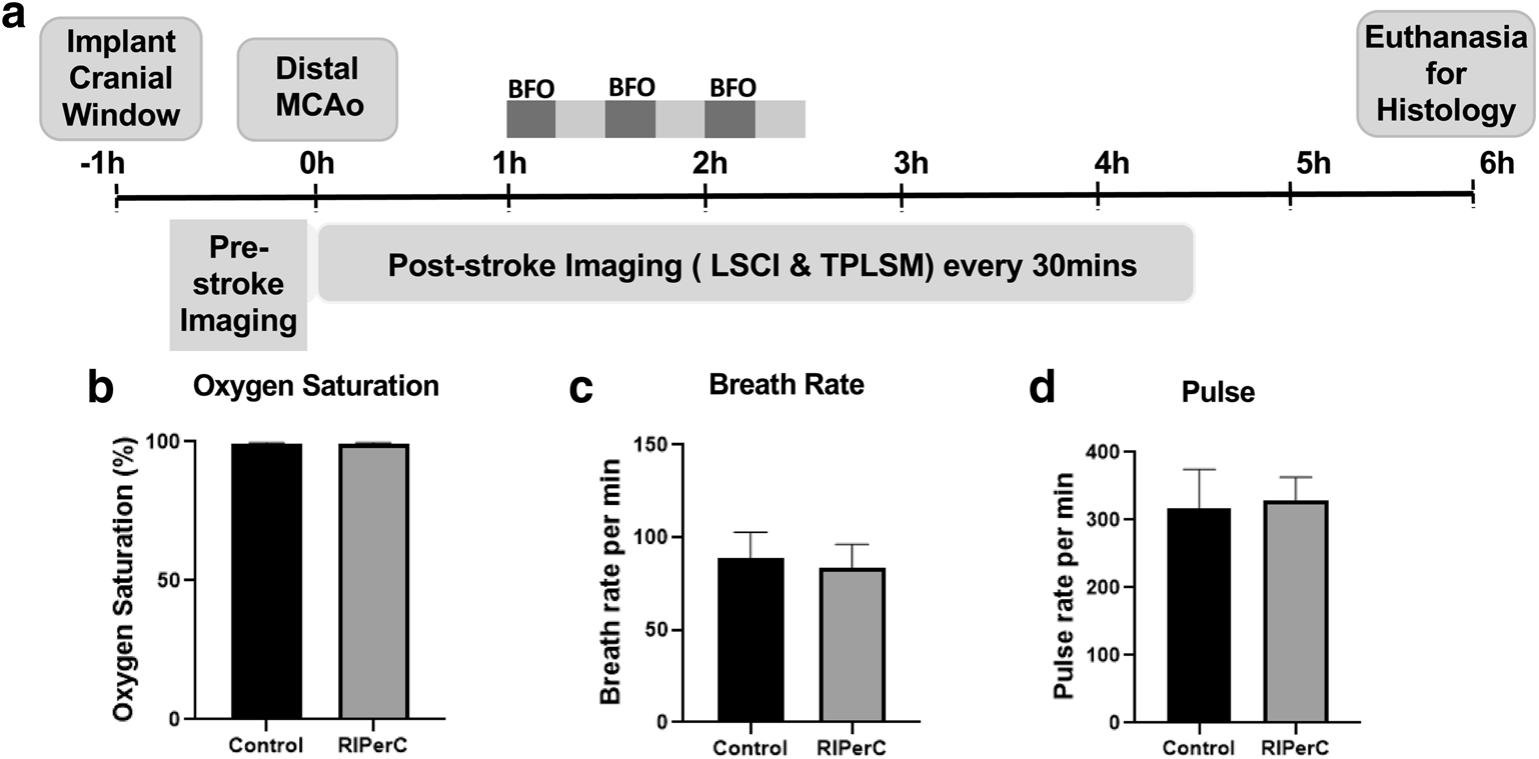
Experimental design and physiological parameters of aged rats treated with RIPerC after dMCAO. (**a**) Experimental timeline (**b**-**d**) Physiological parameters of RIPerC and sham treatment rats during imaging after dMCAO.

**Figure 2:**
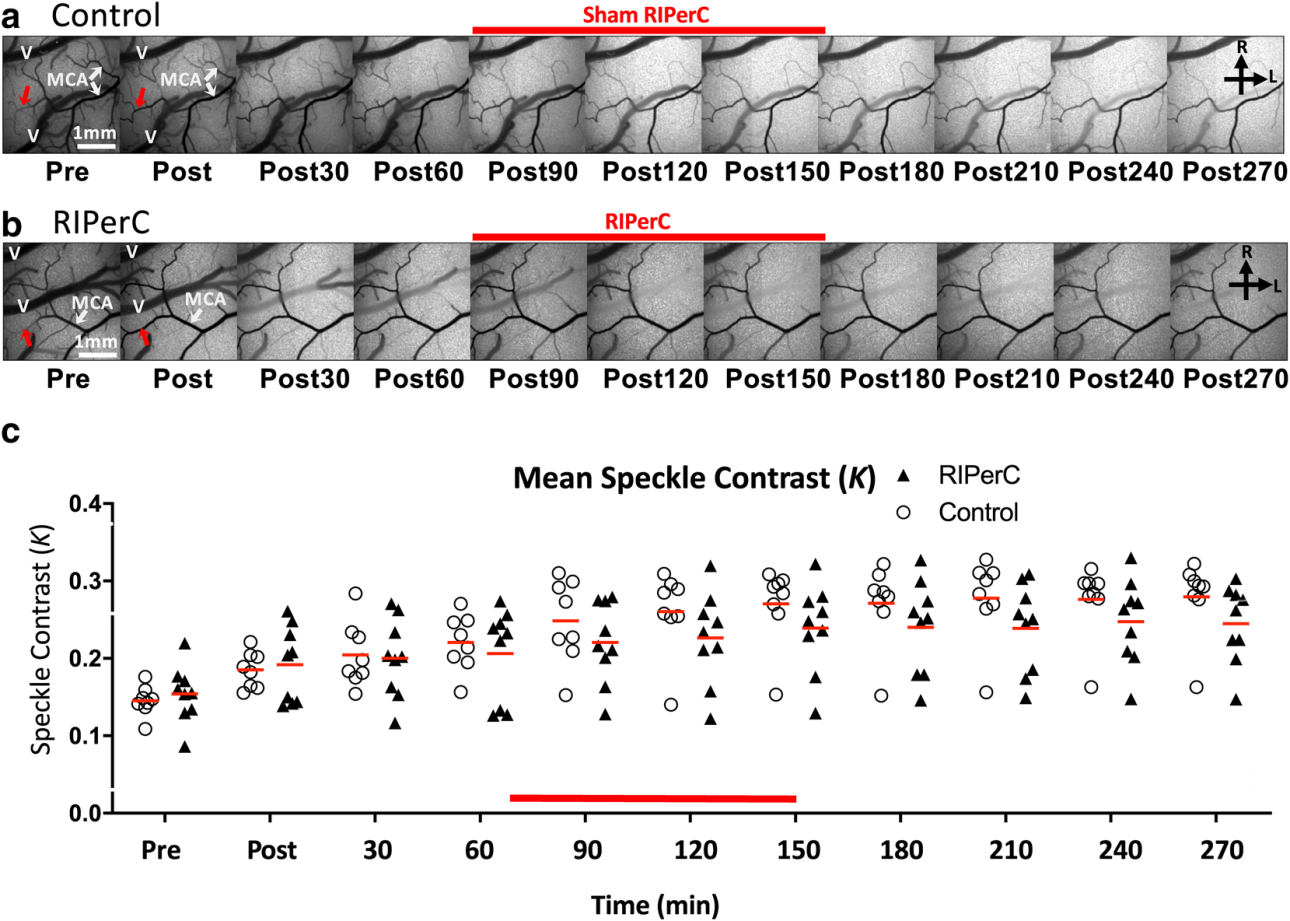
Laser speckle contrast imaging of collateral blood flow. (**a**-**b**) Representative LSCI derived image sequences of speckle contrast showing flow on the cortical surface before and after dMCAO. Images showing flow changes over 270 min (4.5 h) post are illustrated for control (**a**) and RIPerC rats (**b**). Immediately after dMCAO, robust anastomotic connections between distal segments of the ACA and MCA were observed in both groups. (**c**) LSCI revealed an increase of speckle contrast value after dMCAO in both control and RIPerC treated group. Mean speckle contrast values (*K*) are shown in (**c**).

**Figure 3:**
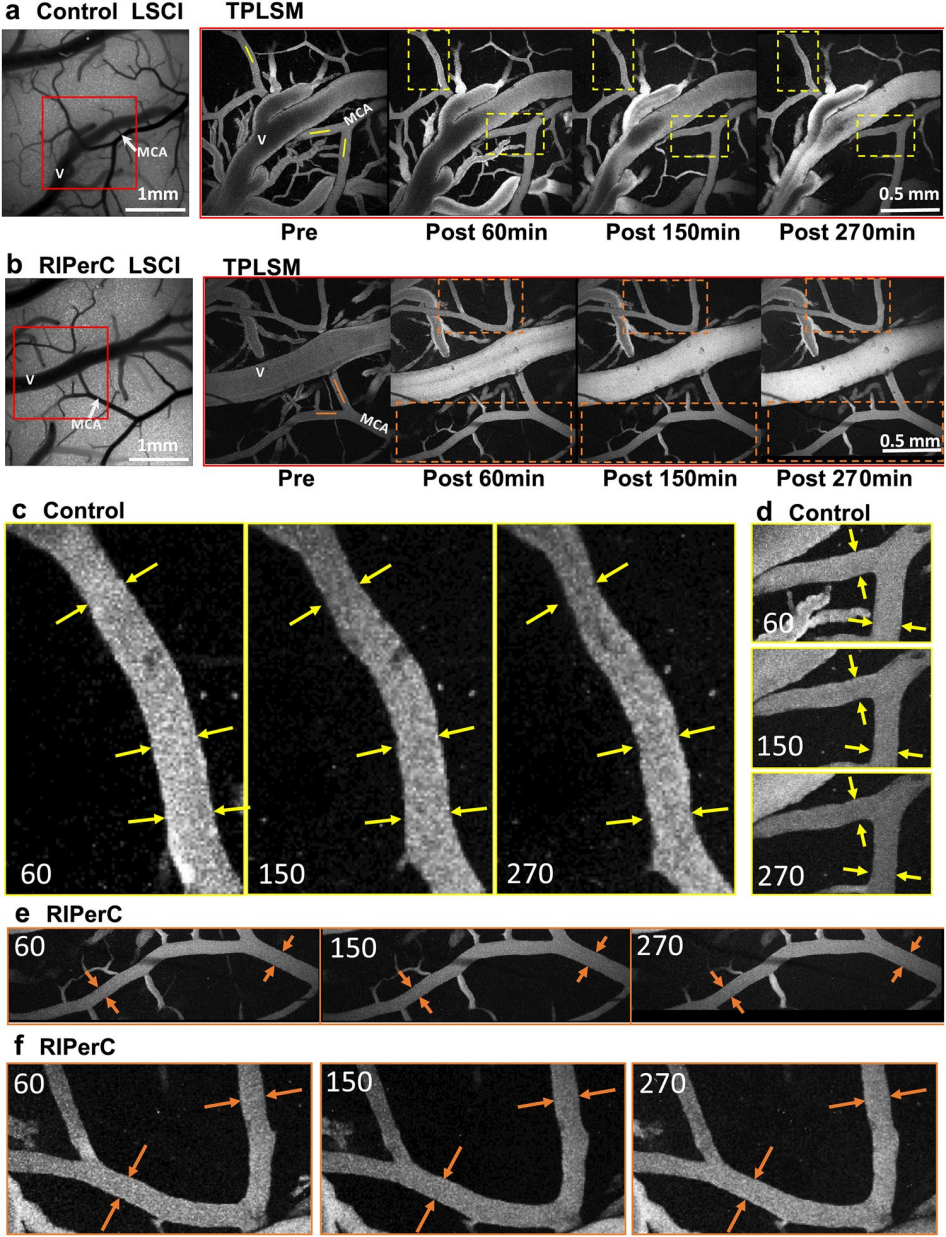
Representative images from TPLSM of collateral blood flow after dMCAO. (**a**,**b**) Representative images of a control rat (**a**) and RIPerC-treated rat (**b**). Panels of TPLSM images on the right show maximum intensity projections from region demarcated with rectangular box in LSCI images. Scale bar, 1 mm. (**c**,**d**) Magnified images showing vessels demarcated by the yellow boxes for the control rat shown in (**a**). Yellow arrows denote vessel diameter 60 min after dMCAO, and are superimposed on the same vessel and region 150 and 270 min after dMCAO. Clear constriction of vessel lumen diameter is apparent in control rats. (**e**,**f**) Magnified images of vessels demarcated by the orange boxes in the RIPerC treated rat in (**b**). Again, orange arrows show vessel diameter at 60 min after dMCAO and are repeated in images acquired 150 and 270 min after ischemic onset. With RIPerC, vessel diameter is maintained throughout the imaging period.

**Figure 4:**
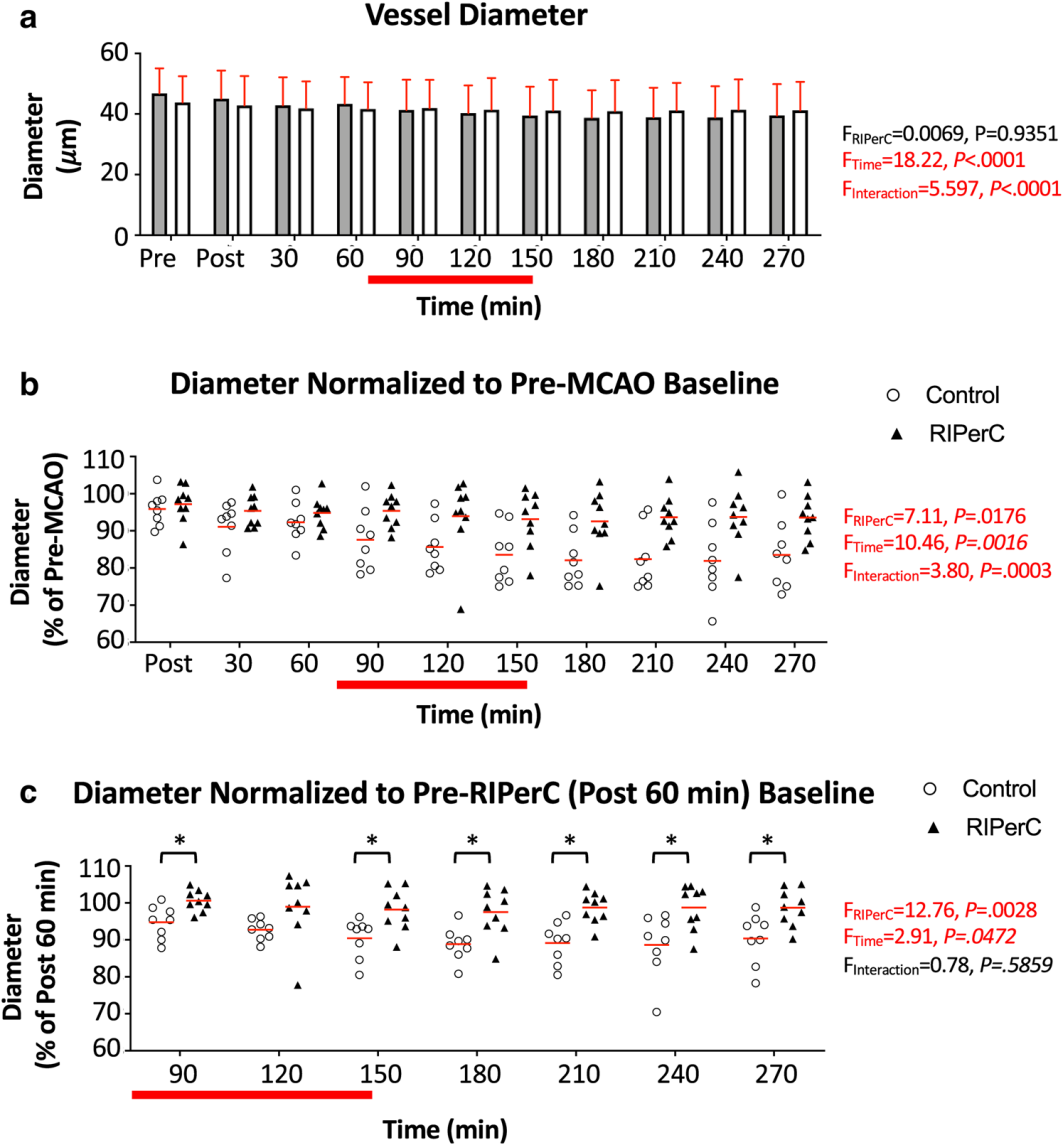
Diameter changes due to dMCAO and RIPerC. (**a**) Mean diameter of vessels measured in control (sham treated) and RIPerC treated aged rats (bars show mean ± SD). Two-way ANOVA confirmed a significant main effect of Time and a significant Time × Treatment interaction (F, *P* values denoted on figure) on pial vessel diameter. To better illustrate diameter changes due to stroke and treatment, (**b**) shows pial diameter data normalized to values measured prior to dMCAO. Individual means for each animal are plotted, and red lines show the group mean. Two-way ANOVA demonstrated a significant main effect of Time and Treatment on pial arteriole diameter, as well as a significant Time × Treatment interaction. To compensate for potential variation in blood flow after dMCAO but prior to treatment, and to isolate treatment effects, (**c**) shows diameters normalized to values measured 60 min after ischemic onset (i.e. post dMCAO but pre-RIPerC or sham treatment). Two-Way ANOVA revealed a significant main effect of Treatment and Time (Time × Treatment interaction n.s.). Holm–Sidak's post hoc comparisons revealed that RIPerC-treated rats had significantly larger diameters (*P* < 0.05) at all-time points after the initiation of the first BFO clamp, with the exception of 120 min after dMCAO.

**Figure 5:**
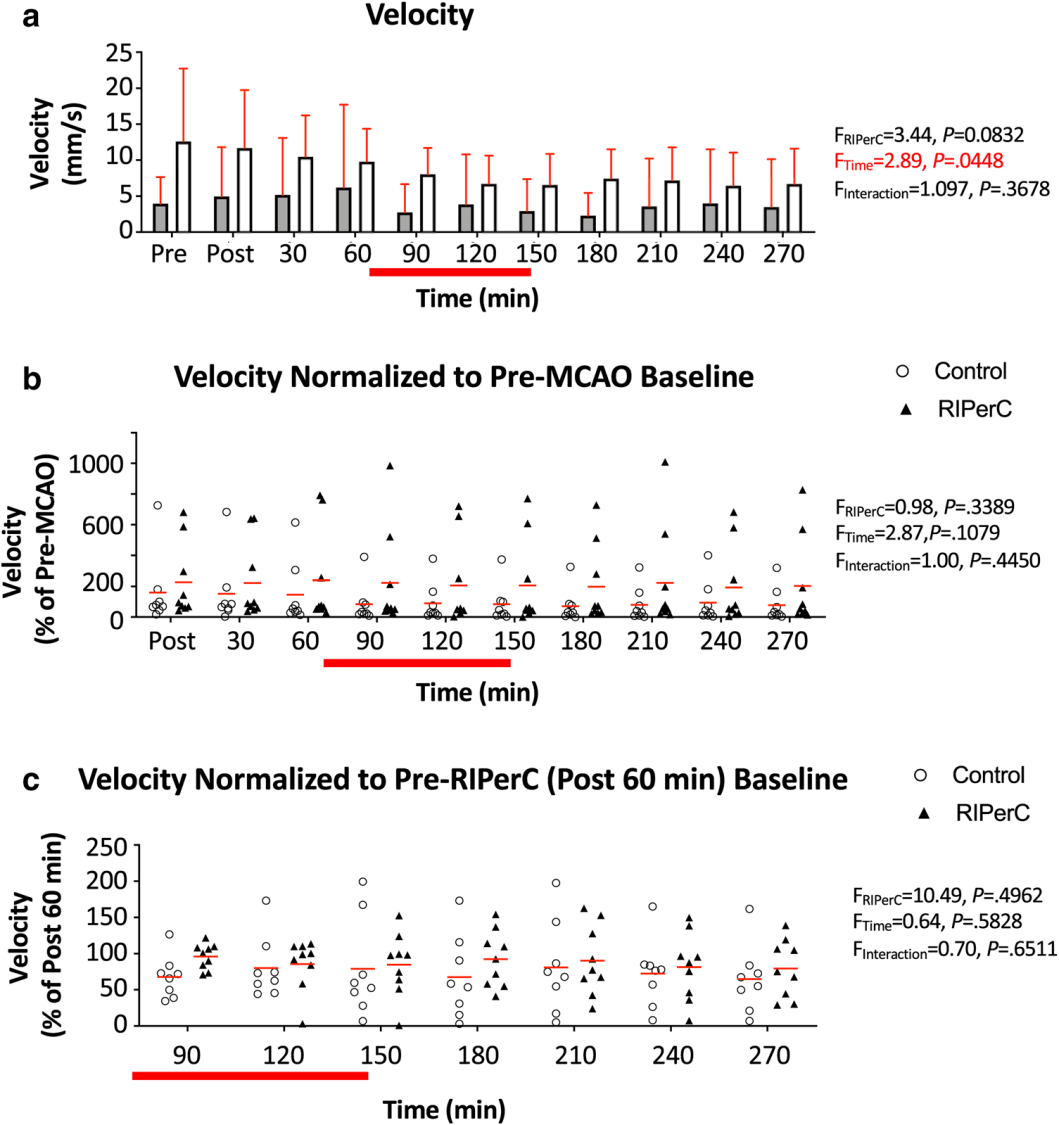
Flow velocity in pial arterioles after dMCAO and RIPerC. (**a**) Mean RBC velocity of pial vessels from control (sham treated) and RIPerC treated aged rats (bars show mean ± SD). Two-way ANOVA confirmed a significant main effect of Time and a trend towards a main effect of Treatment on flow velocity in measured segments. To better illustrate diameter changes due to stroke and treatment, (**b**) shows flow velocity data normalized to values measured prior to dMCAO. (**c**) shows flow velocity normalized to values measured 60 min after ischemic onset (i.e. post dMCAO but pre-RIPerC or sham treatment). Individual means for each animal are plotted, and red lines show the group mean. For both (**b**) and (**c**), two-way ANOVAs demonstrated no significant main effects of Time or Treatment and no significant Time × Treatment interactions.

Pial collateral flow was measured immediately before ischemic onset and for 4.5 h after dMCAO (at intervals of 30 min, Fig. 1a) using LSCI and TPLSM. LSCI and TPLSM were used to create high-spatiotemporal resolution maps of blood flow in pial vessels in the region of ischemia, including measures of regional flow (LSCI, Fig. 2) as well as pial vessel diameter and RBC velocity (TPLSM, Figs. 3–5) (1). Importantly, physiological parameters were not significantly altered by ischemia or repeated imaging (Fig. 1b-d).

### Regional changes in collateral blood flow in aged rats after RIPerC

Figure 2 shows LSCI derived maps of speckle contrast showing flow changes over 270 min (4.5 h) post stroke in aged control rats (Fig. 2a) and aged RIPerC treated rats (Fig. 2b). As previously reported, robust anastomotic connections between the distal segments of MCA and ACA are visible after ischemic onset. These anastomoses are apparent in both groups (red arrows). In aged controls, penumbral flow decreased during the imaging period (as indicated by a consistent reduction in visible pial vessels and increase in speckle contrast brightness during the imaging period (Fig. 2a). Qualitatively, this reduction in flow (increased speckle contrast) appeared less severe in RIPerC treated rats (Fig. 2b). Mean speckle contrast values (*K*) acquired from LSCI data are shown in Fig. 2c. Two-way ANOVA revealed a significant main effect of Time [F _(10,150)_ = 35.67, P < 0.0001), as well as a significant Time × Treatment interaction (F _(10,150)_ = 1.959, *P* = 0.0416); Treatment, n.s.].

### Hemodynamics of collaterals in aged rats after dMCAO

TPLSM was performed to directly assess the luminal diameter and RBC velocity in segments of the distal MCA within or adjacent collateral anastomoses with the ACA in aged rats treated with RIPerC (Fig. 3a) or sham treatment (Controls, Fig. 3b) after distal dMCAO. In Fig. 3, panels at left show LSCI maps before dMCAO. Panels from left to right show TPLSM images from the region demarcated by the red box in the LSCI image acquired before dMCAO then 60 , 150 (during the 3rd cycle of BFO releasing or sham treatment), and 270 min after dMCAO, respectively. Narrowing of distal MCA segments over time after dMCAO was apparent in aged controls, but qualitatively less severe in RIPerC-treated animals. Representative TPLSM images are shown in Fig. 3a,b, with magnified images of the vessels demarcated by boxes in Fig. 3a,b shown in Fig. 3c-f. Arrows in Fig. 3c-f highlight the diameter of these vessels immediately prior to RIPerC (at 60 min after dMCAO). These arrows are then superimposed at the same location on the same vessels imaged at 150 and 270 min after dMCAO for the representative control rat (Fig. 3c,d) and RIPerC treated rat (Fig. 3e,f). Notably, in control rats (Fig. 3c,d) the vessel diameters narrow over time after stroke, such that space between the vessel lumen and the arrows demarcating diameter at 60 min after dMCAO is apparent. Contrasting this, vessel diameters remain stable in RIPerC treated rats (Fig. 3e,f).

Figure 4a shows mean diameter of these pial vessels (within or adjacent ACA-MCA collaterals) at all imaging time points. Mean diameters of the control and RIPerC treated rats at pre-MCAO baseline were were 46.84 ± 2.91 and 43.85 ± 2.88 µm, respectively. Two-way ANOVA demonstrated a significant main effect of Time (F_(3.209, 48.13)_ = 18.22, *P* < 0.0001) and a significant interaction of Time and Treatment (F_(10, 150)_ = 5.597, *P* < 0.0001) on vessel diameter. To isolate diameter changes over time after stroke, vessel diameters were data normalized to baseline values prior to dMCAO (normalized within each vessel then averaged for a mean per animal, Fig. 4b). Two-way ANOVA demonstrated a significant main effect of Time (F_(1.460, 21.90)_ = 10.46, *P* = 0.0016) and Treatment (F_(1,15)_ = 7.106, *P* = 0.0176) on pial arteriole diameter normalized to pre-MCAO baseline, as well as a significant Time × Treatment interaction (F_(9,135)_ = 3.802, *P* = 0.0003). Post hoc comparisons did not identify any significant differences between treatment groups at individual time-points. To further isolate treatment effects and compensate for any potential variation in blood flow after dMCAO but prior to treatment, Fig. 4c shows diameters normalized to values measured 60 min after ischemic onset (but prior to RIPerC or sham treatment). Two-Way ANOVA revealed a significant main effect of Treatment (F_(1,15)_ = 12.76, *P* = 0.0028) and Time (F_(2.886,43.29)_ = 2.905, *P* = 0.0472; Time × Treatment interaction, n.s). Holm–Sidak's post hoc comparisons revealed that RIPerC-treated rats had significantly larger diameters (*P* < 0.05) at all-time points after the initiation of the first BFO clamp, with the exception of 120 min post dMCAO. Thus, consistent with findings in younger adult rats^17^, RIPerC protected against collateral narrowing in aged rats.

Figure 5a shows mean changes in red blood cell (RBC) velocity from the same arterioles corresponding the diameter measurements in Fig. 4. Given their position near or within anastomoses at the distal ends of the MCA territory, flow velocity was variable between vessels before and after dMCAO. However, all segments analyzed exhibited a reversal of flow direction consistent with retrograde flow from ACA to MCA. Two-way ANOVA on flow velocity revealed a significant main effect of Time (F_(3.041, 45.62)_ = 2.892, *P* = 0.0448) and a statistical trend towards an effect of Treatment group (F_(1, 15)_ = 3.444, *P* = 0.0832, reflecting a trend towards a sample with higher velocities before and after stroke in the RIPerC group). Flow velocity normalized to baseline prior to dMCAO and values 60 min after dMCAO are shown in Fig. 5b,c, respectively, to highlight changes due to stroke and treatment. In both cases, there were no significant main effects of Time or Treatment and no significant Time × Treatment interactions.

**Figure 6:**
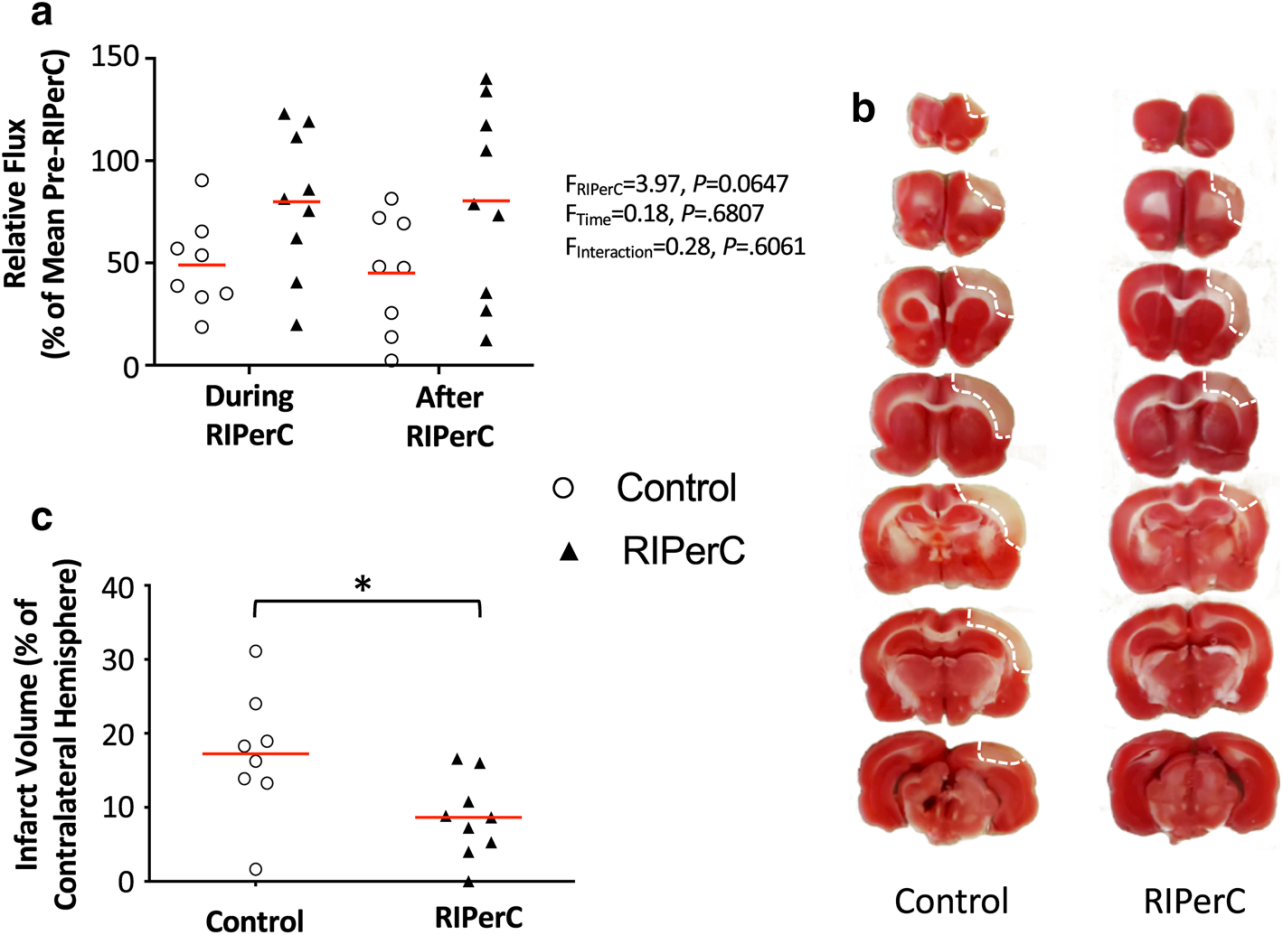
Effects of RIPerC on relative flux and infarct volume in aged rats after dMCAO. (**a**) Relative flux for arteriole segments within or downstream of ACA–MCA collateral anastomoses in RIPerC treated and control rats are shown. Relative flux shows overall flow through these segments during and after RIPerC (or sham treatment), accounting for diameter and velocity measurements, relative to flux prior to treatment. Individual means for each animal are plotted, and red lines show the group mean. RIPerC exhibited a strong statistical trend towards a persistent improvement in flux, with flux relative to pre-treatment values approximately 163% and 178% greater during and after treatment, respectively, in RIPerC treated rats relative to controls. The volume of early ischemic damage from TTC staining for RIPerC and control rats is shown in (**b**) and (**c**). A significant reduction in early infarct was observed in RIPerC treated aged rats.

RBC flux is a critical measure of perfusion, as it is proportional to the oxygen and nutrient carrying capacities of a blood vessel^55,56^. In Fig. 6a, a measure of Relative Flux (see “Materials and methods”) is shown to illustrate changes in flow from post dMCAO baseline during and after RIPerC treatment. Two-way ANOVA demonstrates a statistical trend towards a main effect of Treatment on Relative Flux (F_(1, 15)_ = 3.974, *P* = 0.0647), suggesting that flux was greater in treated rats during and after RIPerC.

### Early ischemic damage

All rats were euthanized at 6 h after dMCAO and stained with TTC to demarcate regions with early ischemic damage (Fig. 6b). As illustrated in Fig. 6c, a significantly smaller volume of early ischemic damage was found in aged rats treated with RIPerC relative to sham treated rats (*P* = 0.0241).

## Discussion

Previous studies suggest that pial collateral blood flow fails over time in aged rats after dMCAO, exacerbating the insufficient perfusion of the penumbra and leading to greater ischemic damage^16^. Collateral failure is also associated with futile recanalization and poor outcome in stroke patients^19,22,57,24^. Clinically, collateral therapeutics that can preserve collateral flow may be able "freeze" penumbra before recanalization via thrombolysis or thrombectomy and thereby reduce stroke severity^58^. Maintaining collateral flow prior to recanalization may be particularly important in aging populations with impaired collateral dynamics.

Several reports suggest that RIPerC can enhance cerebral blood flow in preclinical models of stroke. Studies using regional imaging of cerebral blood flow in mice suggest that RIPerC is effective alone and in combination with i.v. r-tPA in enhancing penumbral flow in young male mice, ovarectomized female mice, and 12-month old male mice^46,47,59,49^. Our previous research with LSCI and TPLSM demonstrate that RIPerC improves pial collateral flow by preventing collateral narrowing in young adult rats after dMCAO^17^. However, this is the first study to use TPLSM to directly record the hemodynamic effects of RIPerC on collaterals in aged animals. A rarefaction of cerebral arterioles and decrease in capillary density occurs with aging in humans, non-human primates, and rodents^60–68^. Advanced age is also associated with narrower lumen diameters and increased tortuosity of cerebral vessels^66,69,70^. These changes in the cerebral circulation increase vascular resistance, thereby reducing tissue perfusion during ischemia and leading to significantly larger regions of infarction^71,9,53,72^. Moreover, vasodilation and vasoreactivity of both peripheral and cerebral circulation may be impaired with aging^73–76^. Combined, these changes could dramatically reduce the compensatory capabilities of the cerebral collateral circulation, and impair the efficacy of collateral therapeutics in aged stroke patients. To be consistent with the Stroke Therapy Academic Industry Roundtable (STAIR) recommendations^77,78^, which were established in an attempt to improve translation of preclinical stroke therapies, it is important then to directly verify the collateral effects of RIPerC in aged animals that better approximate human stroke patients. Our combination of LSCI and TPLSM in aged rats provides high resolution data on the dynamics of collateral connections between the ACA and MCA during MCAO and treatment with RIPerC.

The combination of TPLSM and LSCI in aged rats provided both wide field assessment of penumbral blood flow as well as precise quantification of the diameter of pial vessels and the direction and velocity of blood flow within individual collateral segments. The speckle contrast (*K*) measured with LSCI (Fig. 2) suggested enhancement of blood flow in RIPerC treated rats. More quantitative analyses using TPLSM (Figs. 3–6), confirmed that pial collaterals of aged control rats constrict after dMCAO, reaching approximately 83% of prestroke baseline diameter by 4.5 h after stroke onset. Importantly, consistent with our findings in young adult rats, collateral failure was significantly reduced by RIPerC treatment, with pial arteriole diameter measured at approximately 97% of pre-treatment values 4.5 h hour after dMCAO onset. This increased diameter was associated with a strong statistical trend towards a persistent increase in flux through collaterals in RIPerC treated aged rats (due to diameter changes rather than RIPerC induced changes in flow velocity), and significantly smaller volumes of ischemic damage at 6 h post dMCAO. Notably, our assessment of pial collaterals focused on distal segments of the MCA within or immediately adjacent anastomoses with distal ACA segments. These vessels exhibited flow reversal due to retrograde flow from the ACA to the MCA territory, and in some cases demonstrated dramatic increases in flow velocity following MCAO. However, consistent with our previous studies of aged animals^16^, vessel diameter narrowed over time blood flow velocity was reduced over time in aged control animals. Consequently, flux through these collateral connections fell to ~ 45% of Pre-RIPerC values following the sham treatment period in control animals (as compared to ~ 80% following RIPerC in treated rats). Recently, Zhu et al.^79^ used Doppler optical coherence tomography to demonstrate spatiotemporally dynamic changes in pial collateral flow in young adult rats following distal MCAO and treatment with sensory stimulation. Their data shows significant reductions in flow velocity and flux in ischemic tissue that were stable or increased over time. While there are methodological differences (e.g. different anaesthetics, protocols for inducing MCAO, imaging window preparations, and ages of animals), their findings in 3–4 month old rats are consistent with our previous observation that compensatory increases in flow velocity can maintain stable flow through collaterals in young adult but not aged rats^16^. Notably, in the majority of animals, Zhu et al.^79^ reported a significant subset of MCA branches continued to show anterograde blood flow patterns over time despite severing of the cortical MCA. Moreover, sensory stimulation had protective effects that were greater in the vessels with anterograde flow relative to retrograde flow branches. The authors discuss a model illustrating that flow from a restricted number of collateral connections with the ACA (i.e. retrograde vessels) could provide flow through the vascular network and account for anterograde flow in imaged vessels. This would be consistent with our data: While we report only retrograde flowing vessels, our TPLSM imaging was focused on collateral connections between the most distal branches of the ACA and MCA where flow reversal would be predicted. We would predict that other interconnected vessels downstream of these regions would show anterograde flow as described. Zhu et al.^79^ do not report a narrowing of pial vessels consistent with our data. However, whereas our study focused on pial arterioles within or adjacent ACA/MCA anastomoses at the distal ends of the vascular territories, Zhu et al.^79^ analyzed branches throughout their imaging window. Notably, the mean *radius* at baseline sampled by Zhu et al.^79^ was greater than 60 µm, whereas the mean *diameter* of the vessels measured in our RIPerC and sham treatment groups was 45.26 ± 2.02 µm. Thus, their study provides a comprehensive analysis of changes in flow in larger pial vessels throughout the ischemic region, but might underestimate the dynamics and potentially important contributions of smaller arterioles that constitute the collateral connections.

Our findings of RIPerC in aged rats provide further scientific rationale for exploring translation of RIPerC treatment from bench to bedside. Notably, a transient vasodilatory effect of RIPerC in the cerebral circulation has been reported in humans in clinical studies of subarachnoid hemorrhage^80^. The first randomized trial to examine adjunctive neuroprotective effects of RIPerC to r-tPA treatment in acute stroke patients in the prehospital setting has been conducted^81,82^. This trial found the approach was safe and no intolerable discomfort or adverse events were caused by RIPerC. After adjustment for baseline severity of hypo-perfusion, there was evidence of tissue protection by RIPerC in post hoc MRI data analysis using a voxel based logistic regression method^47,81^. Moreover, significantly lower NIHSS scores and higher frequency of TIA diagnosis were also observed at admission for RIPerC treated patients relative to controls. Remote ischemic conditioning before and after recanalization in patients with large-vessel occlusions of the anterior circulation has also been shown to be safe and feasible preliminary clinical investigations^83,84^. Thus, RIPerC is a promising adjunctive therapy to spare functionally intact brain tissues before recanalization or after treatment.

Several important questions regarding the mechanisms and efficacy of RIPerC as a collateral therapeutic and neuroprotective strategy for ischemic stroke remain. Notably, our study used urethane anaesthetized animals, and it would be ideal define the hemodynamics of RIPerC in unanaesthetized animals. Similarly, while cranial windows allow for stable imaging, use of a thinned skull preparation^85^ could reduce potential confounds due to skull removal and replacement with a glass window. Moreover, it is important to note that most preclinical studies of RIPerC (including ours) induce RIPerC on rodent hind limbs via bilateral femoral ligation^59,49,17^. However, clinical studies generally involve upper limb ischemia via inflation of a blood pressure cuff^82,54,81^. Consistent methods of remote conditioning and consistent periods of ischemia and reperfusion should be used to better validate RIPerC. Additionally, the mechanisms of RIPerC with respect to neuroprotection and collateral dynamics remain to be elucidated. Raised intracranial pressure may contribute to collateral failure^86–90^, and may be accelerated in the aged, and the protective effects of RIPerC on the brain and vasculature may act reduce intracranial pressure to preserve collaterals. However, preliminary studies in clinical populations did not identify an effect of RIPerC on intracranial pressure^84^. Nitric oxide and its metabolites are thought to contribute to protection due to remote ischemic conditioning (RIC) protection in liver and cardiac ischemia. Notably, treated mice have elevated nitrite and nitrate levels in the blood, liver, and heart and the cardioprotective effects of RIC are lost in endothelial nitric oxide synthase (eNOS) knockout mice^95, 91–98^. Plasma nitrite levels also increase after remote ischemic conditioning in healthy human volunteers^95^. The link between nitric oxide and collateral dynamics in cerebral ischemia remains to be further investigated. However, Hoda et al.^49^ found that the mRNA expression of eNOS was dramatically increased in blood vessels after RIPerC during cerebral ischemia, concurrent with increased concentrations of nitric oxide in plasma. It is likely that RIPerC acts through multiple humoural mediators, and further investigation of nitric oxide related effects and novel mediators are required.

Future preclinical models should incorporate reperfusion to better model clinical realities. Increasingly, recanalization is achieved for ischemic stroke patients (particularly for proximal large vessel occlusions), but futile recanalization and the no-reflow phenomenon mean that clinical outcomes do not always match recanalization status. Notably, tissue level reperfusion is a better predictor of outcome than recanalization^99^. A major advantage of TPLSM is resolution of microvascular flow in the capillary bed that reflects tissue level perfusion, and future studies should incorporate transient proximal MCAO models and visualization of flow at the capillary level to better evaluate the efficacy of collateral therapeutics such as RIPerC to induce true reperfusion in ischemic regions.

## Materials and methods

All procedures for collateral blood flow imaging in rats have been previously described^16,17^. Aged Male Sprague–Dawley rats (16–18 months of age) were used (sham treatment: n = 8; RIPerC: n = 9). Prior to experimental procedures, animals were housed in pairs on a 12-h day/night cycle and had access to food and water ad libitum. All procedures conformed to guidelines established by the Canadian Council on Animal Care and were approved by the University of Alberta Health Sciences Animal Care and Use Committee. Methodological and results reporting are consistent with the ARRIVE guidelines for preclinical research^100^. The experimental timeline is illustrated in Fig. 1a.

### Anesthesia and monitoring

An induction chamber with 4–5% isoflurane (in 70% nitrogen and 30% oxygen) was used to induce light anaesthesia prior to intraperitoneal injections of urethane (i.p. 1.25 g/kg, divided into four doses delivered at 30-min intervals). Isoflurane was discontinued after the first urethane injection, and rats remained anaesthetized until euthanasia. Throughout the experiment, temperature was maintained at 36.5–37.5 °C with a thermostatically controlled warming pad. Pulse oximetry (MouseOx, STARR Life Sciences) was used to monitor heart rate, oxygen saturation, and breath rate.

### Cranial window

LSCI and TPLSM were performed through craniotomy based cranial windows. These cranial windows allow for stable vascular imaging in rodents over hours to days^101–104^. After a midline incision, a 5 × 5 mm section of the skull over the distal regions of the right MCA territory was thinned using a dental drill until translucent (repeated flushes with saline are used to dissipate heat). The thinned bone was gently removed, and the dura matter reflected. The cranial window was then covered with a thin layer of 1.3% low melt agarose and sealed with a glass coverslip^16,17,105^.

### dMCAO

A distal model of MCAO was used. To do so, bilateral common carotid artery (CCA) ligation was administered concurrent to ligation of a distal cortical branch of the MCA^16,17^. Distal MCA ligation and imaging protocols were performed by different individuals, and surgeons inducing ischemia were blind to the experimental group for each rat. CCAs were accessed through ventral midline cervical incisions and ligated below the carotid bifurcation (4–0 Prolene sutures). An incision over the right temporalis muscle was performed, and the muscle was gently separated from the bone. A dental drill was used to make a burr hole of 1.5 mm in diameter made through the squamosal bone. Dura was then removed and the exposed distal MCA was isolated with a loose square knot using an atraumatic 9–0 Prolene suture above the rhinal fissure. After pre-stroke imaging, the knot was ligated to induce permanent dMCAO.

### LSCI

LSCI was used to create high spatial and temporal resolution maps of collateral blood flow on the cortical surface^106,18,107,108^. Rats were secured in ear bars on a custom-built stereotaxic plate under a Leica SP5 MP laser scanning microscope. The cortex was illuminated with a Thorlabs LDM 785S laser (20 mW, 785 nm) at approximately 30° incidence. 101 sequential images (1,024 × 1,024 pixels) were acquired at 20 Hz (5 ms exposure time) during each imaging time point. All processing and analysis of laser speckle images were performed using ImageJ software (NIH) by a blinded experimenter. Maps of speckle contrast were made by determining the speckle contrast factor *K* for each pixel in an image. *K* is the ratio of the standard deviation to the mean intensity (*K* = σ_s_/I) in a small (5 × 5 pixels) region of the speckle image (26–28). Plots of *K* show maps of blood flow with darker vessels illustrating faster blood flow velocity. For quantification of penumbral flow, *K* was measured in a contiguous ROI consisting of an 800 × 800 pixel square positioned to include the distal MCA and ACA segments.

### TPLSM

A Leica SP5 MP TPLSM and Coherent Chameleon Vision II pulse laser tuned to 800 nm was used for TPLSM in rats with blood plasma labelled with fluorescein isothiocyanate–dextran (70,000 MW, Sigma-Aldrich) injected (0.3 mL (5% (w/v) in saline, 0.2 mL supplements as required) via the tail vein^16,17,104^. Z-stacks through the first 0.15 mm of cortical tissue were acquired using a 10 × water dipping objective (Leica HCX APO L10 × /0.3 W). Vessel diameter measurements were made from maximum intensity projections of these stacks using an ImageJ plug-in (full-width at half-maximum algorithm)^109^. Diameter measurements were made along three distal segments of the MCA that included or were immediately adjacent anastomotic connections with the ACA (i.e. vessels that were predicted in pre-MCAO baseline imaging to exhibit retrograde flow from ACA to MCA territory after dMCAO). Red blood cell (RBC) velocity was determined using line scans along the lumen of these three arteriole segments over a length of approximately 100 pixels at scan rates of 1200 Hz. RBC velocity was determined from line scan images by calculating the slope of streaks^104,102^. Notably, while segments for analysis were selected prior to MCAO, all vessels included in our analyses demonstrated a reversal of flow direction confirming retrograde flow from ACA collaterals. Mean diameter and velocity measurements are illustrated in Figs. 4 and 5, respectively. In each case, raw measurements are shown (Figs. 4a,5a) in addition to values normalized to Pre-MCAO baseline (Figs. 4b,5b and to Pre-RIPerC baseline (normalized to values at 60 min Post-MCAO, Figs. 4c,5c). Normalizations were performed within each vessel. That is, for each diameter or velocity measurement pertaining to an individual vessel, measurements at all time points following MCAO were divided by Pre-MCAO baseline measurement to better visualize changes in diameter or velocity due to stroke then treatment (Figs. 4b,5b). Similarly, raw measurements were also normalized relative to the final measurement prior to commencing RIPerC treatment (60 min Post-MCAO) to best visualize changes in diameter or velocity induced by treatment (Figs. 4c,5c). Normalizations were performed within each individual vessel then averaged within animals to provide a mean value for each animal. Finally, we calculated a measure of “Relative Flux” to provide an index in changes in overall flow due to treatment (Fig. 6a). RBC flux calculations provide an overall measure of flow through each vessel incorporating lumen diameter and flow velocity, and can be calculated using the following equation:

$${\text{Flux}} = \left( {\pi /{8}} \right)\left( {d^{2} } \right)\left( v \right)$$

where v is the RBC velocity along the central axis of the vessel, and d is the vessel diameter. To calculate Relative Flux, we first calculated RBC flux for each vessel at all timepoints after the induction of dMCAO. The mean flux was then calculated for the period prior to treatment (the mean of Post-MCAO, 30 min, and 60 min Post-MCAO flux values), during RIPerC (mean of 90, 120, and 150 min Post-MCAO flux) and Post-RIPerC (180, 210, 240, and 270 min Post-MCAO flux). Figure 6a shows Relative Flux during and after RIPerC (i.e. mean values during these periods were normalized to the Pre-RIPerC values for each vessel, with the mean per animal plotted and used in statistics).

### RIPerC via bilateral femoral occlusion (BFO)

BFO was performed as previously described^17^. Femoral arteries were dissected from accompanying veins and nerves below the groin ligaments. RIPerC was initiated 60-min after dMCAO by occluding and releasing the femoral arteries bilaterally with vascular clamps for 3 cycles (each occlusion or release lasted for 15 min). Control rats received a sham surgery with equivalent anesthesia and arterial isolation but did not receive BFO.

### Triphenyl tetrazolium chloride staining

All rats were euthanized 6 h after induction of the dMCAO. The brains were rapidly removed and sliced into seven coronal, 2 mm sections using a brain matrix, then incubated in 2% 2,3,5-triphenyltetrazolium chloride (TTC) solution at 37 °C for assessment of mitochondrial dehydrogenase activity. Tissue damage was assessed in digital images of TTC-stained tissue by a blinded experimenter using ImageJ (NIH) software. Volume of tissues showing early ischemic damage is expressed as a percentage of hemisphere. These measures were calculated for each tissue slice using the indirect method^110,111^ to control for tissue distortion due to edema using the following equation.

Volume of ischemic damage (%hemisphere) = [**Σ(A**_**C**_ **−** **A**_**NI**_**)/Σ(A**_**C**_**)]** * **100.**

where A_C_ is the area of the hemisphere contralateral to stroke in a given tissue slice and A_NI_ is the area of the non-injured tissue (i.e. non-ischemic tissue that stains red using TTC) in the ipsilateral (stroke affected) hemisphere of the same slice.

### Statistical analysis

Statistical analyses were performed using Graph Pad Prism (GraphPad software, San Diego, CA, US). After confirming a normal distribution using Kolmogorov–Smirnov test, a mixed model two-way analysis of variance (ANOVA) with Time and Treatment (RIPerC or Sham) as factors was used to evaluate LSCI measures (speckle contrast *K*) and TPLSM measures (vessel diameter, RBC velocity, and Relative Flux). Post hoc comparisons were performed using Holm–Sidak multiple comparisons test. Statistical significance was defined as *P* < 0.05. A statistical trend was defined as *P* > 0.05 < 0.10. Volumes of ischemic tissue infarct (% of contralateral hemisphere) and physiological parameters (pulse, respiratory rate and oxygen saturation) between treatment groups were compared using an unpaired Student's *t* test. Sample sizes estimates were based on data from previous LSCI and TPLSM experiments in our laboratory^16,17,105,18^.

## Ethics approval

University of Alberta Animal Care and Use Committee (AUP0000361).
